# Supplementary material for: Deep learning to estimate lung disease mortality from chest radiographs
Source: Nat Commun. 2023 May 16;14:2797. doi: 10.1038/s41467-023-37758-5 (PMC10188525; doi:10.1038/s41467-023-37758-5)
Supplement: Supplementary file 1 — Supplementary Information [file 41467_2023_37758_MOESM1_ESM.pdf]

Deep learning to estimate lung disease mortality from chest radiographs

Jakob Weiss, MD, Vineet K. Raghu, PhD, Dennis Bontempi, David C. Christiani, MD, MPH, Raymond H. Mak, MD, Michael T. Lu, MD, MPH, Hugo J.W.L. Aerts, PhD

I Supplementary Tables

- **Supplementary Table 1:** Patients demographics and baseline risk factors of never and ever smokers in PLCO
- **Supplementary Table 2:** Model architectures and hyperparameters for each of the 20 ensemble models
- **Supplementary Table 3:** Summary of lung disease ICD codes
- **Supplementary Table 4:** Sex-specific survival models developed in the control (no radiograph) arm of PLCO (N = 77,444)

II Supplementary Figures

- **Supplementary Figure 1:** Association between CXR Lung-Risk and baseline clinical risk factors as well as radiology findings in PLCO and NLST
- **Supplementary Figure 2:** Association between CXR Lung-Risk and baseline clinical risk factors in BLCS patients
- **Supplementary Figure 3:** Independent testing of the CXR Lung-Risk model in ever smokers and never smokers of PLCO to estimate lung disease mortality
- **Supplementary Figure 4:** Independent testing of the CXR Lung-Risk model in PLCO stratified by sex to estimate lung disease mortality
- **Supplementary Figure 5:** Independent testing of the CXR Lung-Risk model in PLCO stratified by chronological age to estimate lung disease mortality
- **Supplementary Figure 6:** Independent testing of the CXR Lung-Risk model in the PLCO testing dataset limited to ever smokers and in NLST (all ever smokers) to estimate lung cancer-specific mortality
- **Supplementary Figure 7:** Independent testing of the CXR Lung-Risk model in NLST stratified by sex to estimate lung disease mortality
- **Supplementary Figure 8:** Independent testing of the CXR Lung-Risk model in NLST stratified by chronological age to estimate lung disease mortality
- **Supplementary Figure 9:** Correlation and comparison of CXR Lung-Risk vs. Lung-Age in BLCS patients
- **Supplementary Figure 10:** Correlation and association of CXR Lung-Risk and FEV1 in BLCS patients
- **Supplementary Figure 11:** Concordance index for individual deep learning models and the ensemble model
- **Supplementary Figure 12:** Consort diagram of the study cohorts

Supplementary Tables

Patients demographics and baseline risk factors of never and ever smokers in PLCO

Variables	PLCO			
	Entire dataset	Never smokers	Ever smokers	p
N	10,155	4650	5505	
Age (years)	62.4±5.4	62.6±5.4	62.1±5.3	<0.001
CXR Lung-Risk	63.0±5.5	61.5±4.4	64.2±6.0	<0.001
Sex (m)	51.6% (5232/10,155)	41.5% (1931/4650)	60.0% (3301/5505)	<0.001
Race				
Non-Hispanic White	86.8% (8810/10,155)	86.2% (4009/4650)	87.2% (4801/5505)	
Non-Hispanic Black	6.0% (611/10,155)	5.8% (268/4650)	6.2% (343/5505)	
Hispanic	2.0% (202/10,155)	1.9% (87/4650)	2.1% (115/5505)	
Other	5.2% (532/10,155)	6.2% (286/4650)	4.5% (246/5505)	0.001
Obesity (BMI>30 kg/m2)	24.2% (2460/10,155)	23.3% (1083/4650)	25.0% (1377/5505)	0.04
Smoking				
Never	45.8% (4650/10,155)	100% (4650/4650)	NA	
Former	43.2% (4391/10,155)	NA	79.8% (4391/5505)	
Current	11.0% (1114/10,155)	NA	20.2% (1114/5505)	<0.001
Pack years	19.2±27.7	0	35.5±29.0	<0.001
Diabetes	7.1% (718/10,155)	6.6% (305/4650)	7.5% (413/5505)	0.08
Hypertension	32,8% (3333/10,155)	33.6% (1562/4650)	32.2% (1771/5505)	0.13
Past Myocardial infarction	8.8% (893/10,155)	6.2% (290/4650)	11.0% (603/5505)	<0.001
Past Stroke	2.4% (246/10,155)	1.9% (88/4650)	2.9% (158/5505)	0.001
Past Cancer	4.1% (414/10,155)	3.9% (182/4650)	4.1% (232/5505)	0.45
Radiology findings				
Nodule	16.6% (1684/10,155)	14.0% (652/4650)	18.7% (1032/5505)	<0.001
Atelectasis	0.2% (18/10,155)	0.09% (4/4650)	0.4% (14/5505)	0.04
Pleural fibrosis	8.0% (811/10,155)	5.6% (259/4650)	10.0% (552/5505)	<0.001
Lung fibrosis	17.2% (1746/10,155)	14.3% (664/4650)	19.7% (1082/5505)	<0.001
COPD/ Emphysema	6.3% (639/10,155)	3.5% (164/4650)	8.6% (475/5505)	<0.001
Lung Opacity	2.6% (259/10,155)	1.9% (90/4650)	3.1% (169/5505)	<0.001

**Cardiac Abnormality**	9.4% (950/10,155)	9.1% (422/4650)	9.6% (528/5505)	0.37
**Lymphadenopathy**	1.2% (121/10,155)	0.8% (39/4650)	1.5% (82/5505)	0.003
**Bone/Chest Wall Lesion**	9.8% (995/10,155)	9.5% (444/4650)	10.0% (551/5505)	0.44
**Lung disease mortality**	5.2% (523/10,155)	1.8% (84/4650)	8.0% (439/5505)	<0.001
**Lung cancer-related mortality**	2.5% (252/10,155)	0.5 % (21/4650)	4.2% (213/5505)	<0.001
**Follow-up in years (IQR)**	17.0 (14.8-19.0)	17.2 (15.4-19.1)	16.8 (14.2-18.8)	<0.001

Supplementary Table 1: Patient demographics and baseline risk factors of PLCO participants stratified by ever vs. never smokers. Radiographic findings were reported by centrally qualified radiologists. As appropriate, the chi-square test, student's t-test or Wilcoxon test were calculated. All p-values are two-sided.

PLCO=Prostate, Lung, Colorectal, Ovarian Cancer Screening Trial; IQR=interquartile range; lung disease mortality=composite endpoint of lung disease-related deaths inducing chronic obstructive pulmonary disease, emphysema, interstitial pulmonary disease and lung cancer.

Model architectures and hyperparameters for each of the 20 ensemble models

Model Number	Batch Size	Epochs	Architecture	Learning Rate(s)	Mixup Data Augmentation	LASSO Coefficient
**LASSO Intercept**						49.85
**0**	16	58	inceptionv4	0.003938972	0	-1.16
**1**	32	41	inceptionv4	7.299E-03, 5.546E-04	0	0
**2**	64	56	inceptionv4	5.474E-03, 2.504E-04	0	2.00
**3**	128	50	tiny	0.006511004	0	-1.67
**4**	32	56	tiny	5.230E-03, 2.272E-04	1	1.80
**5**	64	64	inceptionv4	4.522E-03, 2.682E-04	1	-0.49
**6**	256	66	inceptionv4	1.759E-03,3.753E-05	1	0
**7**	32	53	resnet34	1.353E-03,5.631E-05	1	0
**8**	16	40	inceptionv4	0.008946	0	0
**9**	16	55	resnet34	4.423E-03,9.210E-04	0	-0.22
**10**	256	51	inceptionv4	7.781E-03,2.395E-04	0	0.98
**11**	128	43	inceptionv4	7.310E-03,5.340E-04	0	0
**12**	128	59	resnet34	0.002123776	0	-0.12
**13**	128	49	inceptionv4	1.920E-03,7.070E-04	1	0
**14**	64	43	tiny	5.309E-03,1.857E-04	0	2.95
**15**	32	61	inceptionv4	0.009207081	0	2.79
**16**	256	46	tiny	0.005282977	0	-0.84
**17**	32	43	tiny	3.581E-03,9.715E-04	0	2.71
**18**	64	56	resnet34	2.528E-03,3.341E-04	1	0.98
**19**	256	48	inceptionv4	5.941E-03,3.818E-04	1	0

Supplementary Table 2: Model architectures and hyperparameters for each of the 20 ensemble models. Models with multiple learning rates were trained using a two-phase approach where the model was trained for 50% of epochs with a high learning rate, then fine-tuned with a lower learning rate. Batch size was randomly chosen from the set [32,64,128,256], epochs were chosen uniformly at random from [40,70], architecture was chosen randomly from the set [tiny, resnet34, inceptionv4], and learning rate was chosen uniformly at random from [1e-3, 1e-6]

Summary of lung disease ICD codes

PLCO	
**ICD**	**Disease**
162	Lung cancer
NA	Interstitial pulmonary disease
NA	Emphysema
NA	COPD
**NLST**	
C34	Lung cancer
J841/J849	Interstitial pulmonary disease
J439	Emphysema
J440/J449	COPD

Supplementary Table 3: Summary of ICD codes used for model development and analyses. Codes are provided by the trial codebook and were collected from the death certificate and death review. In PLCO, trial coordinators grouped lung diseases apart from lung cancer into a single category making a further subdivision impossible.

ICD=International classification of diseases

Sex-specific survival models developed in the control (no radiograph) arm of PLCO (N = 77,444)

Risk factor	Coefficient (Male)	p-value (Male)	Coefficient (Female)	p-Value (Female)
**Diabetes**	-1.54	<0.001	-3.51	<0.001
**Age (per 1 yr)**	-0.69	<0.001	-0.68	<0.001
**Obese**	-0.10	0.75	-0.15	0.72
**Underweight**	-6.27	<0.001	-7.19	<0.001
**Hypertension**	-0.65	0.02	-0.97	0.006
**Past MI**	-2.14	<0.001	-2.91	<0.001
**Past Stroke**	-1.47	0.03	-3.35	<0.001
**History of Cancer**	-1.38	0.004	-1.42	0.002
**Pack-Years**	-0.08	<0.001	-0.12	<0.001
**Current smoking**	-10.87	<0.001	-11.20	<0.001
**Former smoking**	-4.07	<0.001	-3.80	<0.001
**Intercept**	83.58		86.53	

Supplementary Table 4: Models were used to estimate age-at-lung-death for those that did not die of a lung-related disease during follow-up. Models were not used for testing dataset results, where only actual, observed lung-related mortality is reported. For example, expected age of dying of a lung-related disease for a 66 year-old male with hypertension and past-MI (myocardial infarction) would be given by:
Current Age + Intercept for men – age * 0.64 – coefficient(hypertension) – coefficient(past MI) = 66 + 65.48 – (66*0.64) – 1.71 – 2.78 = 66 + 18.75 = 84.75

Supplementary Figures

a) PLCO

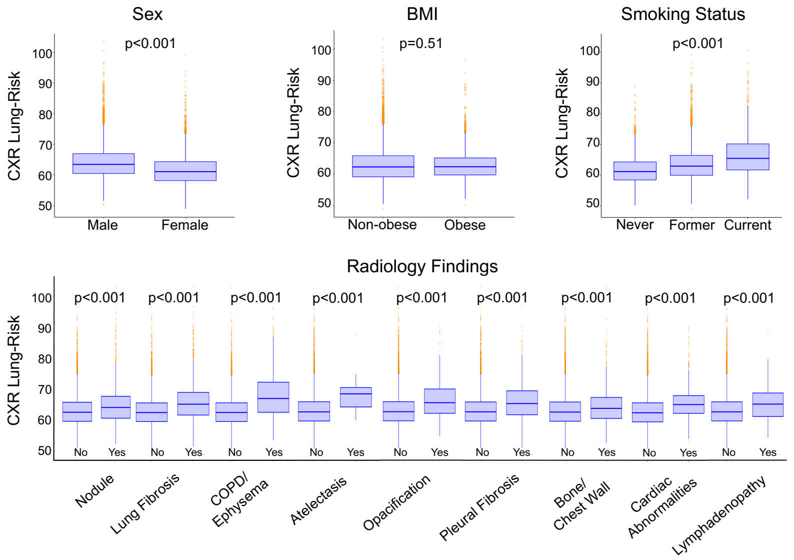

b) NLST

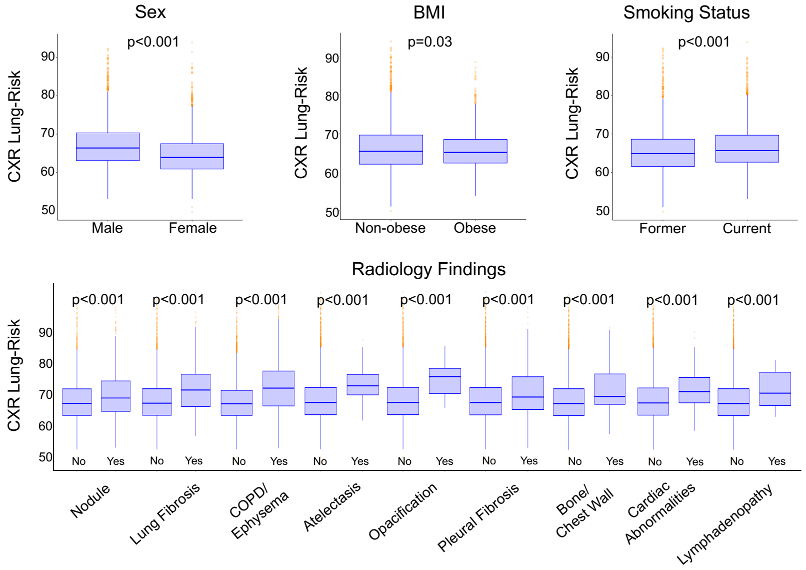

Supplementary Figure 1: Association between CXR Lung-Risk and baseline clinical risk factors as well as radiology findings in PLCO and NLST. a) Association between CXR Lung-Risk and baseline clinical risk factors as well as radiology findings in PLCO (n=10155 independent individuals) and b) NLST (n=5414 independent individuals). CXR Lung-Risk was significantly higher in males, and current/former smokers and if radiologic findings were present. As appropriate, student's t-test, Wilcoxon test or Kruskal wallis test were calculated. All p-values are two-sided. Center line defines median; box limits show interquartile ranges; whiskers show 1.5x interquartile range; points indicate outliers.

PLCO=Prostate, Lung, Colorectal, Ovarian Cancer Screening Trial; NLST=National Lung Screening Trial; BMI=Body Mass Index

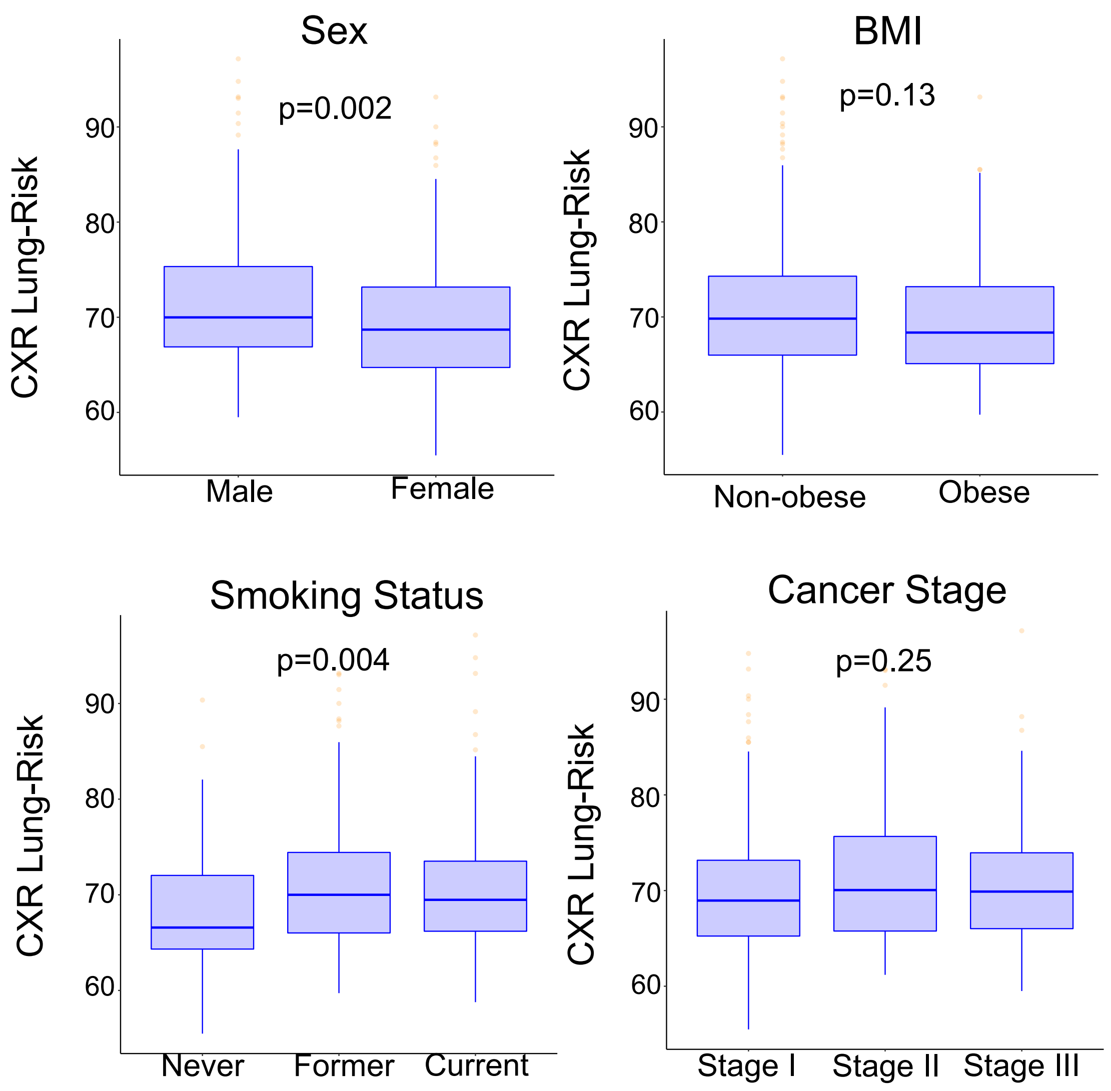

Supplementary Figure 2: Association between CXR Lung-Risk and baseline clinical risk factors in BLCS patients (n=407 independent individuals). CXR Lung-Risk was significantly higher in males, and current/former smokers. As appropriate, student's t-test, Wilcoxon test or Kruskal wallis test were calculated. All p-values are two-sided.Center line defines median; box limits show interquartile ranges; whiskers show 1.5x interquartile range; points indicate outliers.

BLCS=Boston Lung Cancer Cohort; BMI=Body Mass Index

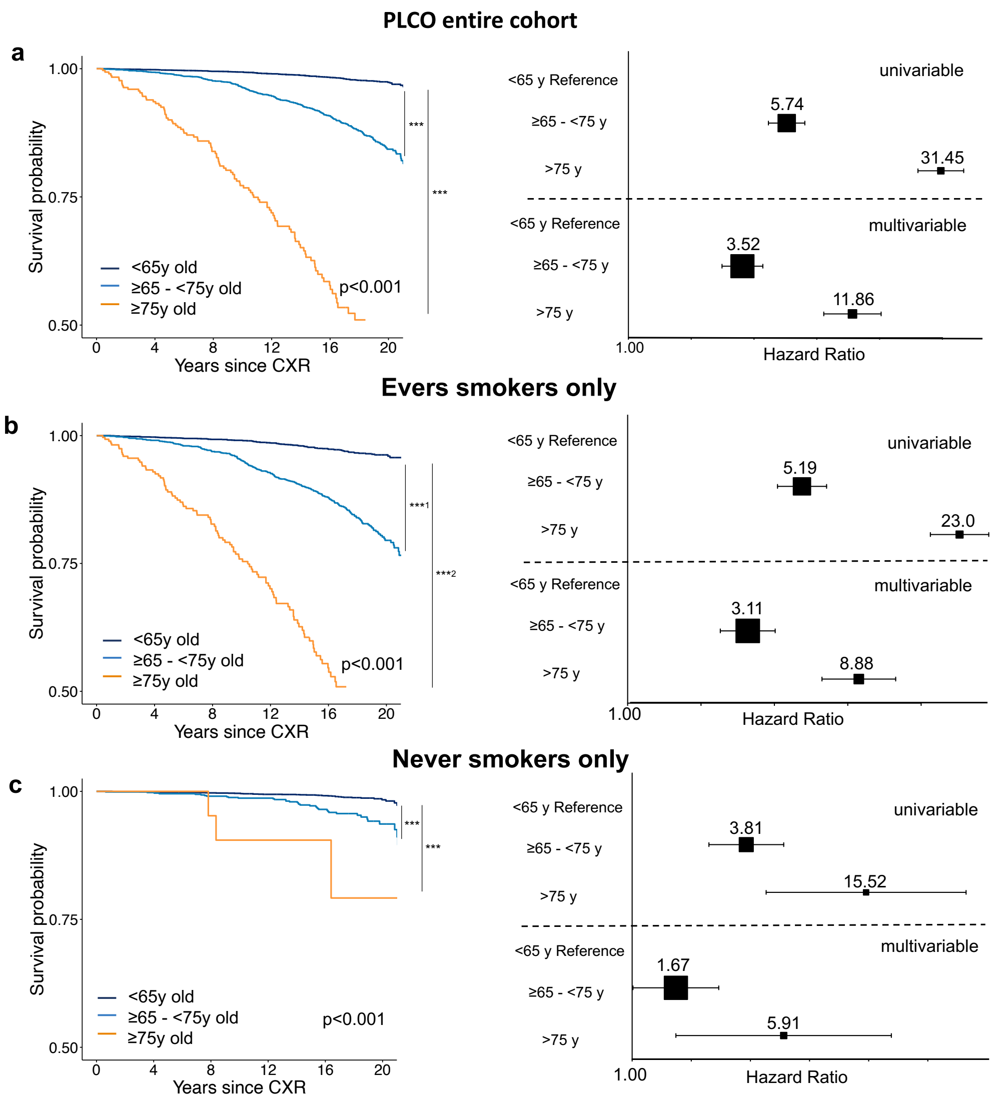

Supplementary Figure 3: Independent testing of the CXR Lung-Risk model in ever smokers and never smokers of PLCO to estimate lung disease mortality. Kaplan-Meier survival analysis shows a graded association between CXR Lung-Risk groups and lung disease mortality in a) the entire PLCO cohort (n=10155 independent individuals) b) ever smokers (n=5505 independent individuals) and c) never smokers (n=4650 independent individuals) of the PLCO testing dataset. Pairwise comparison of survival curves was performed using two-sided Log-Rank tests. P-values are adjusted for multiple comparisons using the Bonferroni-Holm method. Forest plots show univariable and multivariable adjusted hazard ratios (box) with 95% confidence intervals (error bars) for the different CXR Lung-Risk groups. Multivariable models are adjusted for: chronological age, sex, race, smoking status, pack years, body mass index, prevalent diabetes mellitus, hypertension, history of stroke, myocardial infarction, cancer and 9 radiologic findings as described in the methods. Source data are provided as a Source Data file.

***p value<$2*10^{-16}$; ***[1]p value=$3.9*10^{-10}$; ***[2]p value=$1.6*10^{-9}$: CXR=chest radiograph; PLCO=Prostate, Lung, Colorectal, Ovarian Cancer Screening Trial; NLST=National Lung Screening Trial; y=years

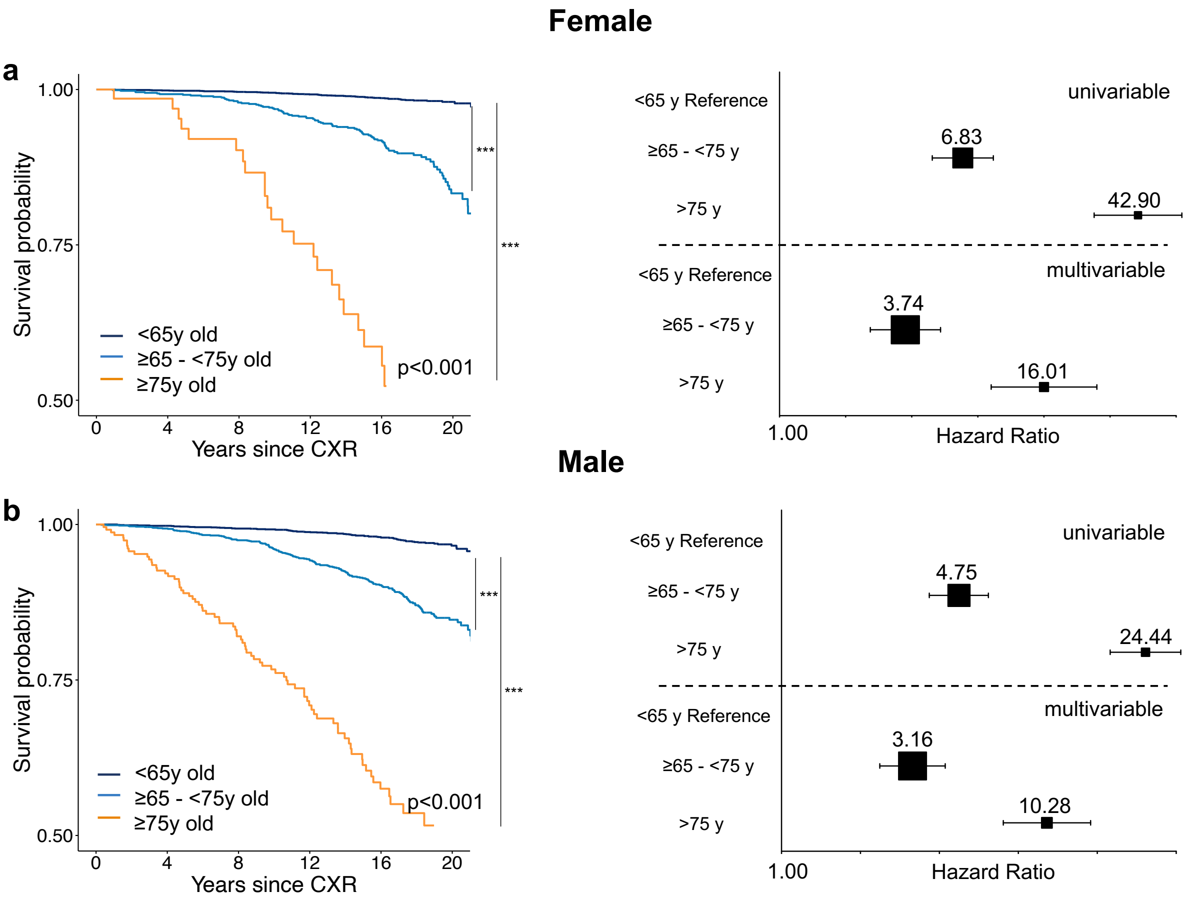

Supplementary Figure 4: Independent testing of the CXR Lung-Risk model in PLCO stratified by sex to estimate lung disease mortality. Kaplan-Meier survival analysis shows a graded association between CXR Lung-Risk groups and lung disease mortality in a) females (n=4923 independent individuals) and b) males (n=5232 independent individuals). Pairwise comparison of survival curves was performed using two-sided Log-Rank tests. P-values are adjusted for multiple comparisons using the Bonferroni-Holm method. Forest plots show univariable and multivariable adjusted hazard ratios (box) with 95% confidence intervals (error bars) for the different CXR Lung-Risk groups. Multivariable models are adjusted for: chronological age, sex, race, smoking status, pack years, body mass index, prevalent diabetes mellitus, hypertension, history of stroke, myocardial infarction, cancer and 9 radiologic findings as described in the methods. Source data are provided as a Source Data file.

***p value<$2*10^{-16}$; CXR=chest radiograph; PLCO=Prostate, Lung, Colorectal, Ovarian Cancer Screening Trial; y=years

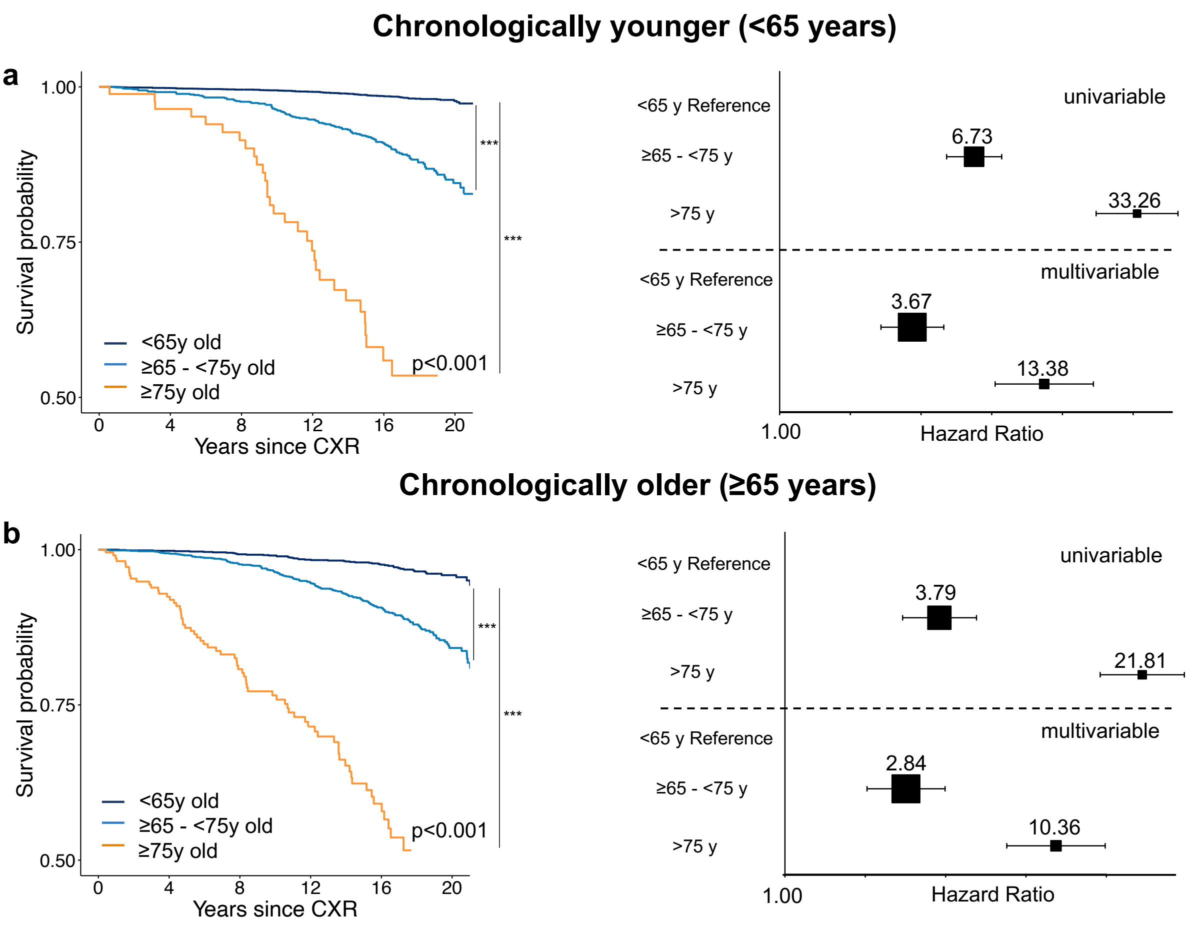

Supplementary Figure 5: Independent testing of the CXR Lung-Risk model in PLCO stratified by chronological age to estimate lung disease mortality. Kaplan-Meier survival analysis shows a graded association between CXR Lung-Risk groups and lung disease mortality in a) chronologically younger (<65 years old; n=6704 independent individuals) and b) chronologically older (≥65 years old; n=3451 independent individuals). Pairwise comparison of survival curves was performed using two-sided Log-Rank tests. P-values are adjusted for multiple comparisons using the Bonferroni-Holm method. Forest plots show univariable and multivariable adjusted hazard ratios (box) with 95% confidence intervals (error bars) for the different CXR Lung-Risk groups. Multivariable models are adjusted for: chronological age, sex, race, smoking status, pack years, body mass index, prevalent diabetes mellitus, hypertension, history of stroke, myocardial infarction, cancer and 9 radiologic findings as described in the methods. Source data are provided as a Source Data file.

***p value$<2*10^{-16}$; CXR=chest radiograph; PLCO=Prostate, Lung, Colorectal, Ovarian Cancer Screening Trial; y=years

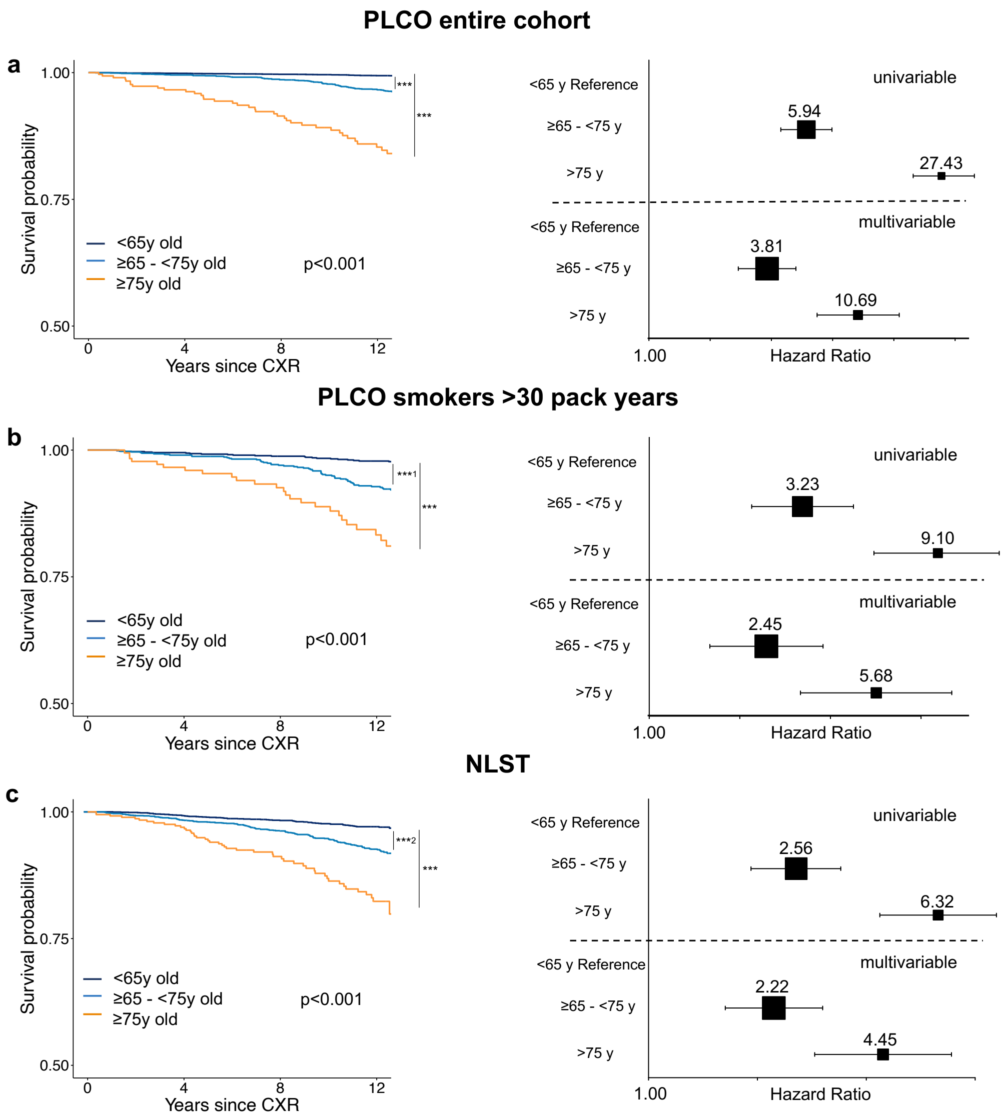

Supplementary Figure 6: Independent testing of the CXR Lung-Risk model in the PLCO testing dataset limited to current or former smokers (quit <15 years ago) with a smoking history of ≥30 pack years and in NLST(all current or former smokers who quit <15 years ago with a smoking history ≥30 pack years) to estimate lung cancer-specific mortality. Kaplan-Meier survival analysis shows a graded association between CXR Lung-Risk groups and lung cancer-specific mortality in a) the entire PLCO cohort (n=10155 independent individuals) b) in current or former smokers (quit <15 years ago) with a smoking history of ≥30 pack years of the PLCO testing data set (n=2031 independent individuals) and in c) NLST participants (n=5414 independent individuals). Pairwise comparison of survival curves was performed using two-sided Log-Rank tests. P-values are adjusted for multiple comparisons using the Bonferroni-Holm method. Forest plots show univariable and multivariable adjusted hazard ratios (box) with 95% confidence intervals (error bars) for the different CXR Lung-Risk groups. Multivariable models are adjusted for: chronological age, sex, race, smoking status, pack years, body mass index, prevalent diabetes mellitus, hypertension, history of stroke, myocardial infarction, cancer and 9 radiologic findings as described in the methods. Source data are provided as a Source Data file.

***p value<$2*10^{-16}$; ***[1]p value=$6.6*10^{-10}$; ***[2]p value=$2.9*10^{-11}$; CXR=chest radiograph; PLCO=Prostate, Lung, Colorectal, Ovarian Cancer Screening Trial; NLST=National Lung Screening Trial; y=years

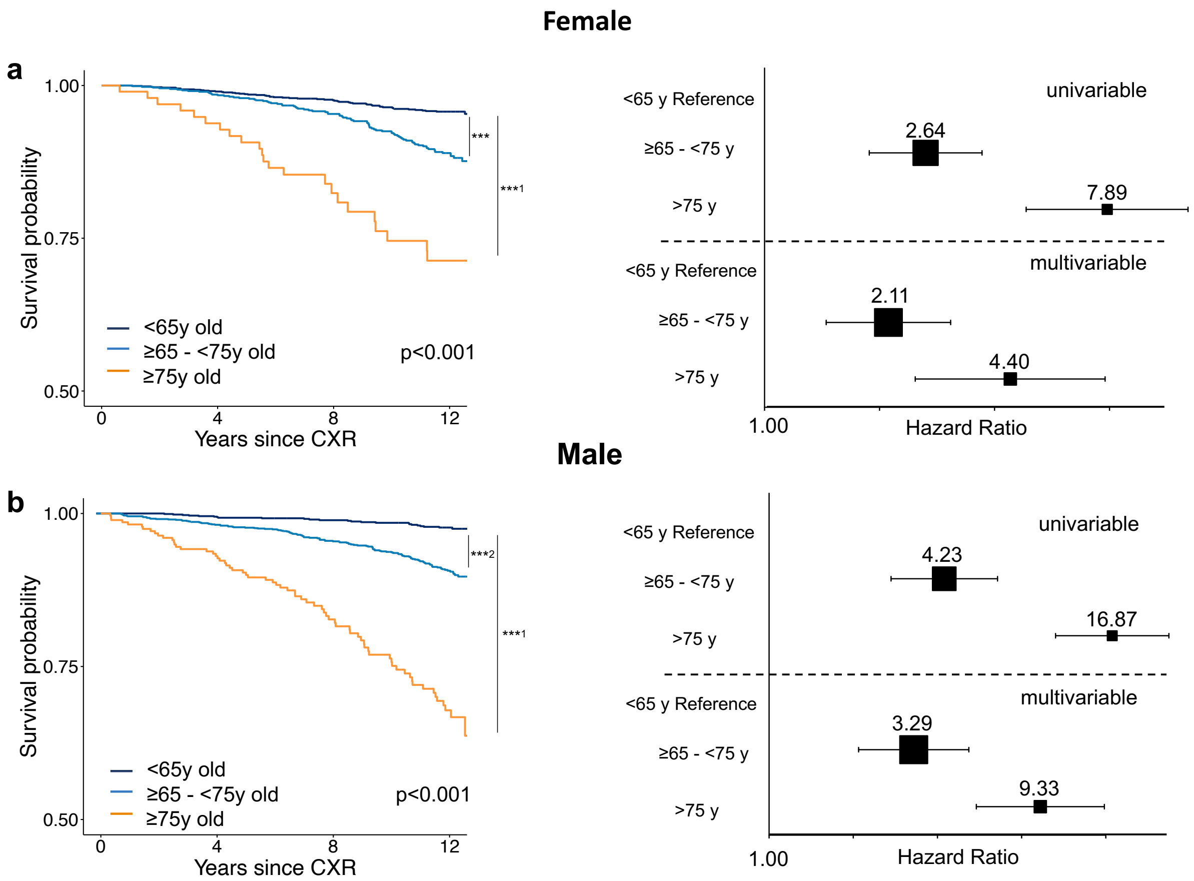

Supplementary Figure 7: Independent testing of the CXR Lung-Risk model in NLST stratified by sex to estimate lung disease mortality. Kaplan-Meier survival analysis shows a graded association between CXR Lung-Risk groups and lung disease mortality in a) females (n=2416 independent individuals) and b) males (n=2998 independent individuals). Pairwise comparison of survival curves was performed using two-sided Log-Rank tests. P-values are adjusted for multiple comparisons using the Bonferroni-Holm method. Forest plots show univariable and multivariable adjusted hazard ratios (box) with 95% confidence intervals (error bars) for the different CXR Lung-Risk groups. Multivariable models are adjusted for: chronological age, sex, race, smoking status, pack years, body mass index, prevalent diabetes mellitus, hypertension, history of stroke, myocardial infarction, cancer and 9 radiologic findings as described in the methods. Atelectasis (for females only) and lung opacity (for males only) were removed from the model because coefficients converged due to too few events. Source data are provided as a Source Data file.

***p value=$7.7*10^{-9}$; ***[1]p value<$2*10^{-16}$; 7b: ***[2]p value=$1.9*10^{-12}$; CXR=chest radiograph; NLST=National Lung Screening Trial; y=years

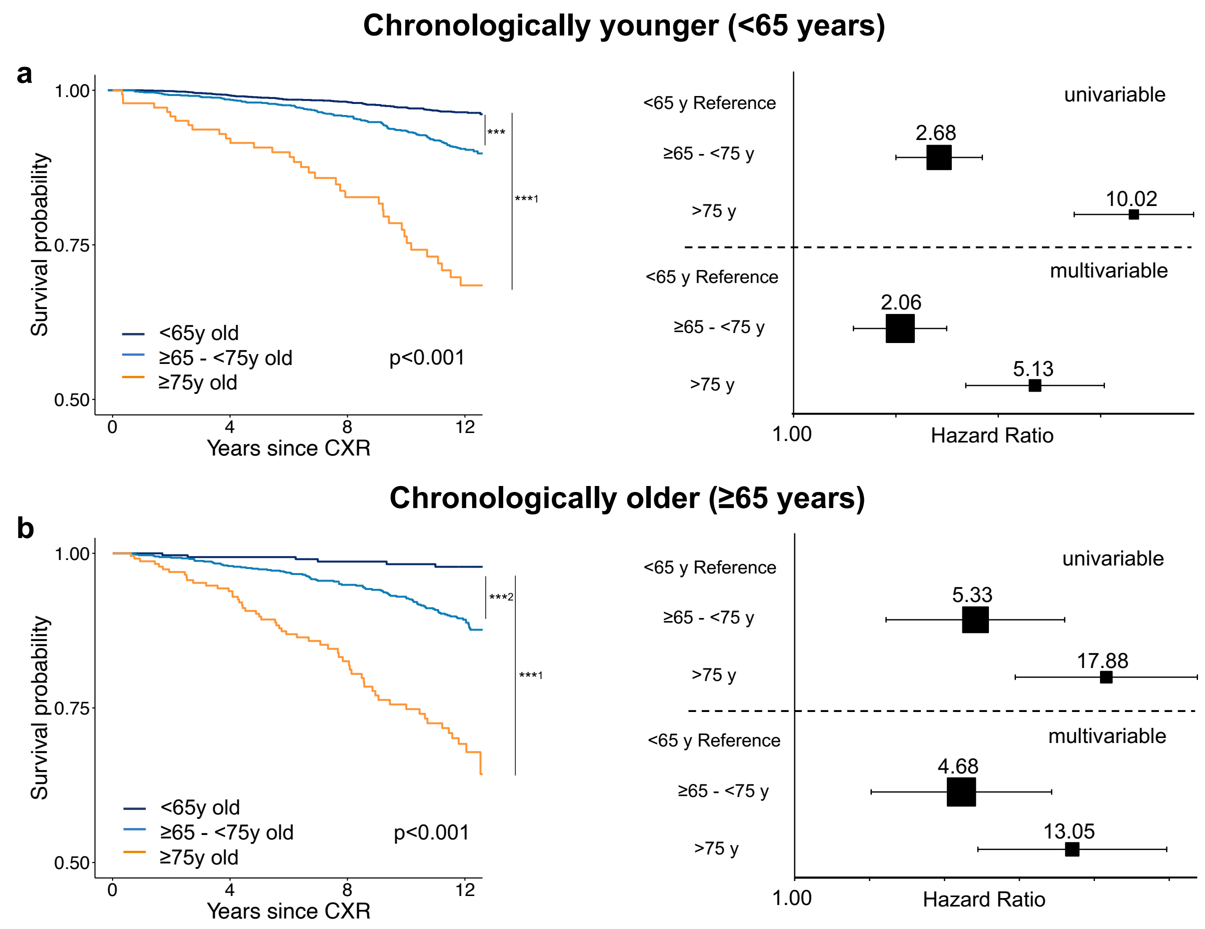

Supplementary Figure 8: Independent testing of the CXR Lung-Risk model in NLST stratified by chronological age to estimate lung disease mortality. Kaplan-Meier survival analysis shows a graded association between CXR Lung-Risk groups and lung disease mortality in a) chronologically younger (<65 years old; n=3854 independent individuals) and b) chronologically older (≥65 years old; n=1560 independent individuals). Pairwise comparison of survival curves was performed using two-sided Log-Rank tests. P-values are adjusted for multiple comparisons using the Bonferroni-Holm method. Forest plots show univariable and multivariable adjusted hazard ratios (box) with 95% confidence intervals (error bars) for the different CXR Lung-Risk groups. Multivariable models are adjusted for: chronological age, sex, race, smoking status, pack years, body mass index, prevalent diabetes mellitus, hypertension, history of stroke, myocardial infarction, cancer and 9 radiologic findings as described in the methods. Atelectasis and bone/chest wall lesions (for chronologically younger only) and lung opacity and lymphadenopathy (for chronologically older only) were removed from the model because coefficients converged due to too few events. Source data are provided as a Source Data file.

***p=6.6*10^{-12}; ***[1]p value<2*10^{-16}; ***[2]=p value=8.5*10^{-6}; CXR=chest radiograph; NLST=National Lung Screening Trial; y=years

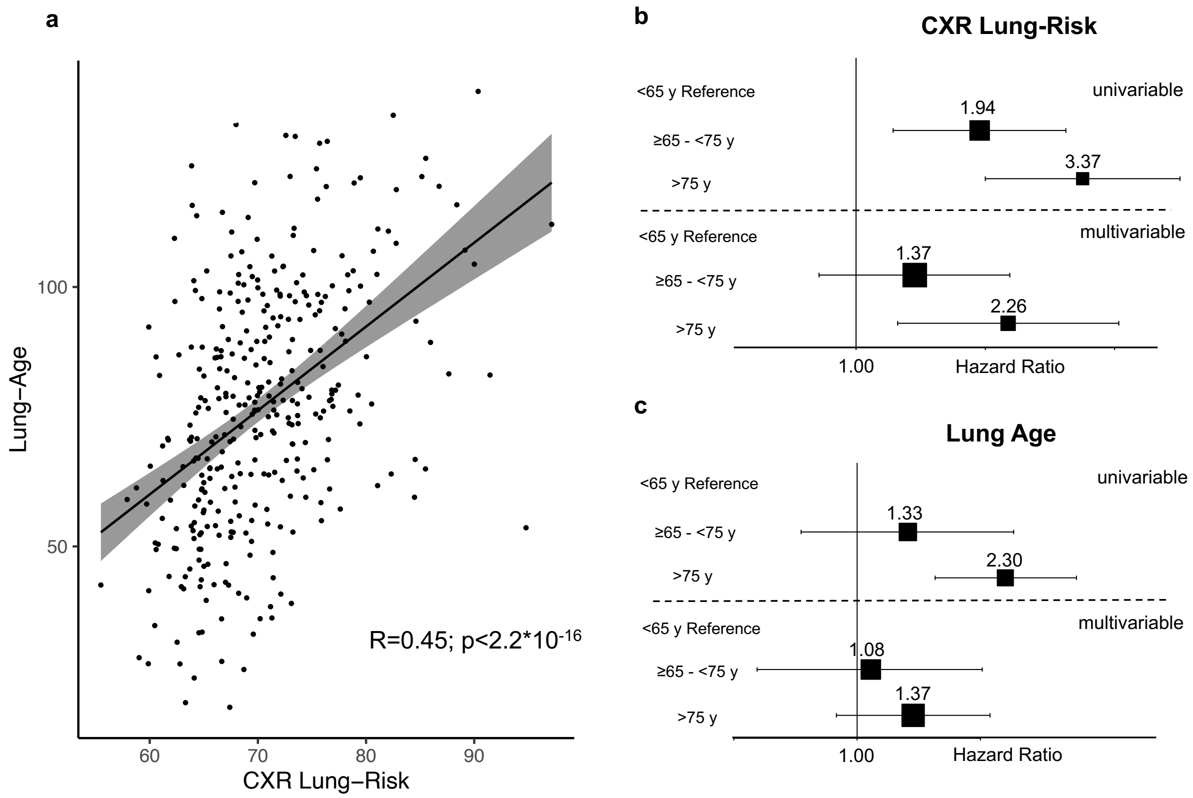

Supplementary Figure 9: Correlation and comparison of CXR Lung-Risk vs. Lung-Age in BLCS patients. a) Correlation between CXR Lung-Risk and traditional Lung-Age in a subset of BLCS patients (n=348 independent individuals) with available lung function tests. Lung-Age was calculated as previously described via a linear regression model with sex, height and FEV1 (l) as input variables. The gray error band represents the 95% confidence interval. b and c) Forest plots show univariable and multivariable adjusted hazard ratios (box) with 95% confidence intervals (error bars) for the different CXR Lung-Risk and conventional Lung-Age groups. Multivariable models are adjusted for: chronological age, sex, race, obesity, smoking status, cancer stage, and treatment. Source data are provided as a Source Data file.

BLCS=Boston Lung Cancer Study; FEV1 (l)=forced expiratory volume in liters during the first second; R=correlation coefficient; y=years

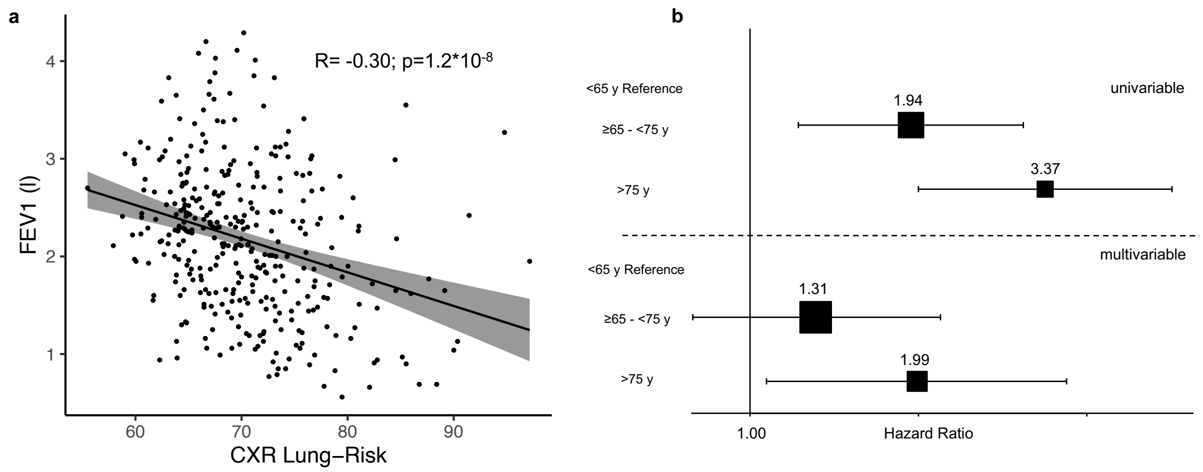

Supplementary Figure 10: Correlation and association of CXR Lung-Risk and FEV1 in BLCS patients. a) Correlation between CXR Lung-Risk and FEV1 in a subset of BLCS patients (n=348 independent individuals) with available lung function tests. The gray error band represents the 95% confidence interval. b) Forest plots show univariable and multivariable adjusted hazard ratios (box) with 95% confidence intervals (error bars) for the different CXR Lung-Risk groups. Multivariable models are adjusted for: chronological age, sex, race, obesity, smoking status, cancer stage, treatment and FEV1. Source data are provided as a Source Data file.

BLCS=Boston Lung Cancer Study; FEV1 (l)=forced expiratory volume in liters during the first second; R=correlation coefficient; y=years

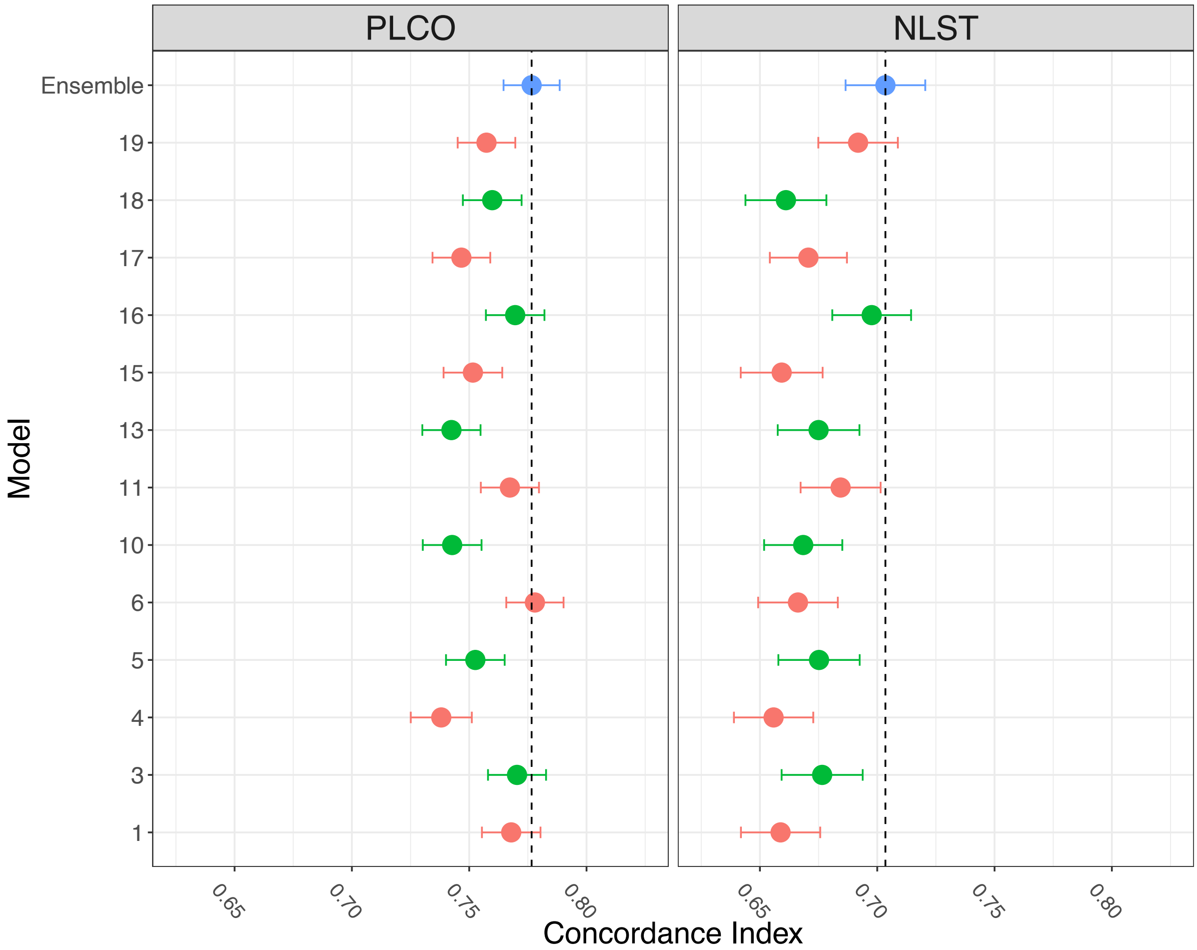

Supplementary Figure 11: Concordance index for individual deep learning models and the ensemble model. Concordance index (points) and standard error (error bars) for individual deep learning models and the ensemble model (blue) for lung disease-related death and lung cancer death in the PLCO (n=10155 independent individuals) and NLST (n=5414 independent individuals) testing datasets.

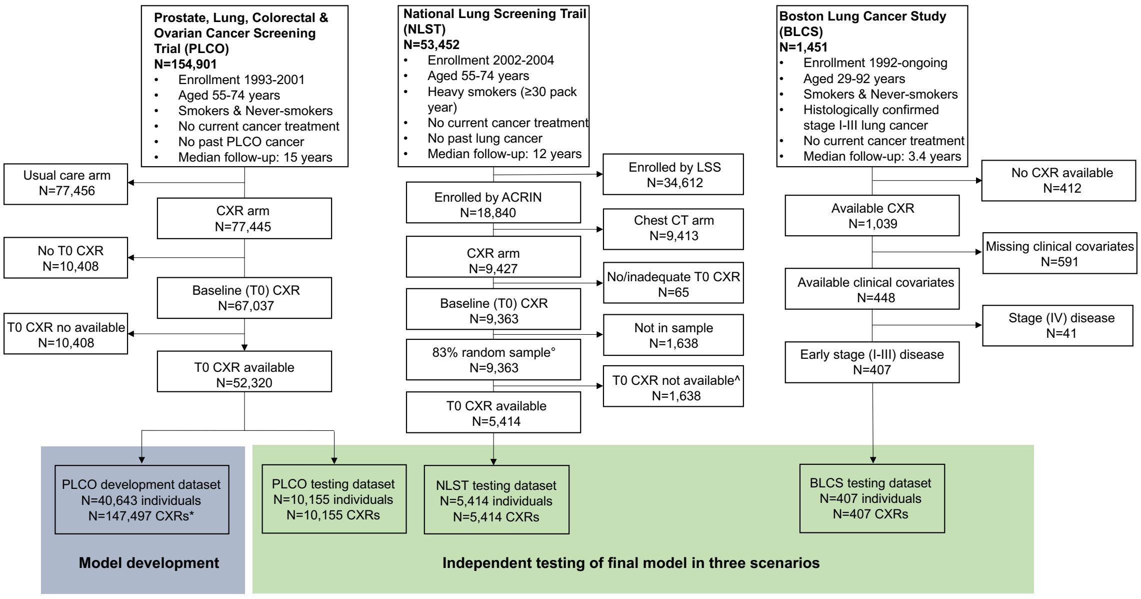

Supplementary Figure 12: Consort diagram of the three study cohorts used for development and independent testing of the CXR Lung-Risk model.

PLCO=Prostate, Lung, Colorectal, Ovarian Cancer Screening Trial; NLST=National Lung Screening Trial, BLCS=Boston Lung Cancer Study; CXR=chest radiograph; T0=baseline CXR; ACRIN=American College of Radiology Imaging Network; LSS=Lung Screening Study group; CT=computed tomography
