# Supplementary material for: Deep learning to estimate lung disease mortality from chest radiographs
Source: Nat Commun. 2023 May 16;14:2797. doi: 10.1038/s41467-023-37758-5 (PMC10188525; doi:10.1038/s41467-023-37758-5)
Supplement: Supplementary file 3 — Reporting Summary [file 41467_2023_37758_MOESM3_ESM.pdf]

nature portfolio

Corresponding author(s): Hugo Aerts

Last updated by author(s): 2023/03/08

Reporting Summary

Nature Portfolio wishes to improve the reproducibility of the work that we publish. This form provides structure for consistency and transparency in reporting. For further information on Nature Portfolio policies, see our Editorial Policies and the Editorial Policy Checklist.

Statistics

For all statistical analyses, confirm that the following items are present in the figure legend, table legend, main text, or Methods section.

n/a	Confirmed	
☐	☒	The exact sample size (*n*) for each experimental group/condition, given as a discrete number and unit of measurement
☐	☒	A statement on whether measurements were taken from distinct samples or whether the same sample was measured repeatedly
☐	☒	The statistical test(s) used AND whether they are one- or two-sided<br>*Only common tests should be described solely by name; describe more complex techniques in the Methods section.*
☐	☒	A description of all covariates tested
☐	☒	A description of any assumptions or corrections, such as tests of normality and adjustment for multiple comparisons
☐	☒	A full description of the statistical parameters including central tendency (e.g. means) or other basic estimates (e.g. regression coefficient) AND variation (e.g. standard deviation) or associated estimates of uncertainty (e.g. confidence intervals)
☐	☒	For null hypothesis testing, the test statistic (e.g. *F*, *t*, *r*) with confidence intervals, effect sizes, degrees of freedom and *P* value noted<br>*Give P values as exact values whenever suitable.*
☒	☐	For Bayesian analysis, information on the choice of priors and Markov chain Monte Carlo settings
☒	☐	For hierarchical and complex designs, identification of the appropriate level for tests and full reporting of outcomes
☐	☒	Estimates of effect sizes (e.g. Cohen's *d*, Pearson's *r*), indicating how they were calculated

Our web collection on statistics for biologists contains articles on many of the points above.

Software and code

Policy information about availability of computer code

Data collection	All the code of the deep learning system including the trained model and the code of the statistical analyses is publicly available on the AIM webpage: https://aim.hms.harvard.edu/cxr-lungrisk. Furthermore, we embedded the deep learning model in an end-to-end pipeline including image preprocessing and model inference that is freely available in a Google Colab cloud-based notebook. This cloud-based instance facilitates future validation studies and allows users with minimal coding proficiency to process a large amount of CXR data without having to install anything on their local node. In the notebook, we describe all the steps of the processing, discuss the different models composing the ensemble and their details, and provide examples. This notebook along with R code to reproduce the statistical analyses can also be found on the AIM webpage: https://aim.hms.harvard.edu/cxr-lungrisk.
Data analysis	All statistical analyses were performed in R (version 3.6.1) using the following packages: survival; survminer, ggplot2; ggpubr, forestplot. The CXR Lung-Risk model was developed using fastai v2.5.3, PyTorch v1.10, and CUDA v11.2. Image convertion (.tif to .png and DICOM to .tif) was perfomred using ImageMagick v6.8.9-9 and DCMTK v3.6.1.

For manuscripts utilizing custom algorithms or software that are central to the research but not yet described in published literature, software must be made available to editors and reviewers. We strongly encourage code deposition in a community repository (e.g. GitHub). See the Nature Portfolio guidelines for submitting code & software for further information.

Data

Policy information about availability of data

All manuscripts must include a data availability statement. This statement should provide the following information, where applicable:

- Accession codes, unique identifiers, or web links for publicly available datasets
- A description of any restrictions on data availability
- For clinical datasets or third party data, please ensure that the statement adheres to our policy

The original PLCO and NLST data cannot be distributed with this publication due to our data use agreements but can be downloaded upon request from the National Cancer Institute (PLCO: https://biometry.nci.nih.gov/cdas/plco/; NLST: https://biometry.nci.nih.gov/cdas/nlst/). The BLCS data are protected under the BLCS study protocol. Access to limited de-identified data can be requested through the BLCS Trial Center for academic non-commercial research purposes only and are subject to review of a project proposal that will be evaluated by a BLCS data access committee. Requests can be made through BLCS webpage (https://www.hsph.harvard.edu/blcs/) or directly by contacting Prof. David Christiani (dchris@hsph.harvard.edu). Requests will be reviewed within two weeks. The CXR Lung-Risk data generated in this study have been deposited on the AIM webpage (https://aim.hms.harvard.edu/cxr-lungrisk) and are available for download to replicate the statistical analysis.

Human research participants

Policy information about studies involving human research participants and Sex and Gender in Research.

Reporting on sex and gender	Sex was self-reported by trial participants in PLCO and NLST. For BLCS patients sex was determined via manual chart review by trained study staff. Analyses in PLCO and NLST are presented for the entire cohort and stratified by sex. in addition, all survival analyses are adjusted for sex as a potential confounder. For BLCS, no sex-stratified analyses were perfomred due to the small sample size not allowing for meaningful conclusions.
Population characteristics	Covariate-relevant population characteristics were as follows for PLCO and NLST: age, sex, race, smoking status, pack years, body mass index, prevalent diabetes mellitus, hypertension, history of stroke, myocardial infarction, and cancer. For BLCS patients, the following covariate were considered age, sex, race, obesity, smoking status, cancer stage (I-III), and treatment (surgery only vs. adjuvant treatment) and FEV (l) = forced expiratory volume in liters in 1 second)
Recruitment	PLCO and NLST participants were recruited according to the trail inclusion criteria. BLCS patients were included if they were referred to the Mass General Brigham Healthcare system for cancer treatment. BLCS results may be bias as it only included patients from a single academic healthcare system in Massachusetts, USA.
Ethics oversight	Secondary use of the investigated study cohorts was approved by the Mass General Brigham institutional review board.

Note that full information on the approval of the study protocol must also be provided in the manuscript.

Field-specific reporting

Please select the one below that is the best fit for your research. If you are not sure, read the appropriate sections before making your selection.

☒ Life sciences ☐ Behavioural & social sciences ☐ Ecological, evolutionary & environmental sciences

For a reference copy of the document with all sections, see nature.com/documents/nr-reporting-summary-flat.pdf

Life sciences study design

All studies must disclose on these points even when the disclosure is negative.

Sample size	A common design in maschine learning studies is to use a data split for model developement and testing. This approach was also applied in the current study with a data split of 80% training and 20% testing). To investigate and test whether the output of the developed CXR Lung-Risk model provides reliable risk estimates of lung disease mortality, the model was independently tested in two held-out datasets (NLST and BLCS) without any retraining.
Data exclusions	Individuals with missing/incomplete a priori defined clinical covariates and/or missing/corrupeted imaging data were excluded.
Replication	Statistical analyses were successfully replicated by a second person.
Randomization	This is an observational study, in which we investigated the association between CXR Lung-Risk and patient characteristics. For model development, a random sample of 80% of individuals enrolled in the intervention arm was used including chest radiographs from all timepoints to avoid sampling bias. The 20% of the training dataset was reserved for hyperpameter tuning. For model development, each radiograph exam was used as an independent sample; for testing, only baseline radiographs defined as the initial radiograph obtained at the enrollment exam were used. Thereafter, the model was locked and applied to the entire NLST and BLCS as independent testing datasets with no additional retraining to investigate generalizablity of the model. To account for the observational study design we adjusted for relevant covariates (e.g. age, smoking status, lung cancer stage) in the statistical analyses.

Blinding	Blinding was not relevant to this retrospective analysis as no intervention was investigated and compared to a control group.

Reporting for specific materials, systems and methods

We require information from authors about some types of materials, experimental systems and methods used in many studies. Here, indicate whether each material, system or method listed is relevant to your study. If you are not sure if a list item applies to your research, read the appropriate section before selecting a response.

Materials & experimental systems

n/a	Involved in the study	
☒	☐	Antibodies
☒	☐	Eukaryotic cell lines
☒	☐	Palaeontology and archaeology
☒	☐	Animals and other organisms
☐	☒	Clinical data
☒	☐	Dual use research of concern

Methods

n/a	Involved in the study	
☒	☐	ChIP-seq
☒	☐	Flow cytometry
☒	☐	MRI-based neuroimaging

Clinical data

Policy information about clinical studies

All manuscripts should comply with the ICMJE guidelines for publication of clinical research and a completed CONSORT checklist must be included with all submissions.

Clinical trial registration	PLCO (NCT00047385); NLST (NCT01696968)
Study protocol	https://cdas.cancer.gov/plco/; https://biometry.nci.nih.gov/cdas/nlst/
Data collection	PLCO: PLCO was a multicenter randomized controlled trial of chest radiography for cancer screening in asymptomatic individuals aged 55-74 years enrolled at 10 US sites from 1993 through 2001.<br>NLST: NLST was a randomized controlled trial that enrolled heavy smokers (≥30 pack years) aged 55-74 years for lung cancer screening via chest CT vs. chest radiograph at 21 US sites from 2002 through 2004.<br>BLCS: The BLCS dataset included 407 patients from the Boston Lung Cancer Study (BLCS), which is an ongoing multicenter observational epidemiologic cohort registry of patients with histologically confirmed lung cancer. For this study, only patients with early stage (I-III) and a diagnosis between 2004-2016 were included.
Outcomes	The primary endpoint of this study was a composite of lung disease mortality including lung cancer, interstitial pulmonary disease, emphysema, and COPD; the secondary endpoint was lung cancer-specific mortality. All cause-specific deaths were based on ICD-9 codes provided in the PLCO and NLST trials. For BLCS, endpoints were verified by manual chart review and available for lung cancer-specific mortality only.
